# Smaller Body Size, Early Postnatal Lethality, and Cortical Extracellular Matrix-Related Gene Expression Changes of *Cyfip2*-Null Embryonic Mice

**DOI:** 10.3389/fnmol.2018.00482

**Published:** 2019-01-04

**Authors:** Yinhua Zhang, Hyojin Kang, Yeunkum Lee, Yoonhee Kim, Bokyoung Lee, Jin Yong Kim, Chunmei Jin, Shinhyun Kim, Hyun Kim, Kihoon Han

**Affiliations:** ^1^Department of Neuroscience, College of Medicine, Korea University, Seoul, South Korea; ^2^Department of Biomedical Sciences, College of Medicine, Korea University, Seoul, South Korea; ^3^Division of National Supercomputing, KISTI, Daejeon, South Korea; ^4^Department of Anatomy, College of Medicine, Korea University, Seoul, South Korea

**Keywords:** *Cyfip2*-null mice, Embryo, body size, postnatal lethality, extracellular matrix

## Abstract

Cytoplasmic FMR1-interacting protein 2 (CYFIP2) is a key component of the WAVE regulatory complex (WRC) which regulates actin polymerization and branching in diverse cellular compartments. Recent whole exome sequencing studies identified *de novo* hotspot variants in *CYFIP2* from patients with early-onset epileptic encephalopathy and microcephaly, suggesting that CYFIP2 may have some functions in embryonic brain development. Although perinatal lethality of *Cyfip2*-null (*Cyfip2*^−/−^) mice was reported, the exact developmental time point and cause of lethality, and whether *Cyfip2*^−/−^ embryonic mice have brain abnormalities remain unknown. We found that endogenous *Cyfip2* is mainly expressed in the brain, spinal cord, and thymus of mice at late embryonic stages. *Cyfip2*^−/−^ embryos did not show lethality at embryonic day 18.5 (E18.5), but their body size was smaller than that of wild-type (WT) or *Cyfip2*^+/−^ littermates. Meanwhile, at postnatal day 0, all identified *Cyfip2*^−/−^ mice were found dead, suggesting early postnatal lethality of the mice. Nevertheless, the brain size and cortical cytoarchitecture were comparable among WT, *Cyfip2*^+/−^, and *Cyfip2*^−/−^ mice at E18.5. Using RNA-sequencing analyses, we identified 98 and 72 differentially expressed genes (DEGs) from the E18.5 cortex of *Cyfip2*^+/−^ and *Cyfip2*^−/−^ mice, respectively. Further bioinformatic analyses suggested that extracellular matrix (ECM)-related gene expression changes in *Cyfip2*^−/−^ embryonic cortex. Together, our results suggest that CYFIP2 is critical for embryonic body growth and for early postnatal survival, and that loss of its expression leads to ECM-related gene expression changes in the embryonic cortex without severe gross morphological defects.

## Introduction

The Cytoplasmic FMR1-interacting protein (CYFIP1 and CYFIP2) family is a critical component of the heteropentameric WAVE regulatory complex (WRC) which regulates actin polymerization and branching in diverse cellular compartments (Abekhoukh and Bardoni, 2014; Lee et al., 2017). Despite their high sequence homology at the protein level (88% identity and 95% similarity) (Schenck et al., 2001), several lines of evidence indicate that CYFIP1 and CYFIP2 have distinct and non-complementable functions *in vivo* (Cioni et al., 2018). For example, both *Cyfip1*- and *Cyfip2*-null mice are lethal at different developmental stages. Specifically, *Cyfip1*-null (*Cyfip1*^−/−^) embryos die before embryonic day 9.5 (E9.5) (Chung et al., 2015). In the case of *Cyfip2*^−/−^ mice, perinatal lethality was reported (Kumar et al., 2013; Han et al., 2015), but the exact developmental time point and cause of lethality, and whether *Cyfip2*^−/−^ embryos have molecular or morphological brain abnormality remain unknown.

Clinically, variants of *CYFIP1* have been associated with neurodevelopmental and neuropsychiatric disorders, including autism spectrum disorders, intellectual disability, and schizophrenia (Abekhoukh and Bardoni, 2014). Although the genetic associations between *CYFIP2* and brain disorders are relatively unknown, two recent whole exome sequencing studies identified *de novo* hotspot variants of *CYFIP2* (at the Arg87 residue) in patients diagnosed with West syndrome (Nakashima et al., 2018; Peng et al., 2018). The variants may disrupt the inhibitory interaction between CYFIP2 and WAVE in the WRC, leading to aberrant activation of the WRC and downstream actin polymerization (i.e., gain-of-function effects on the WRC) (Nakashima et al., 2018). The West syndrome is characterized by early-onset epileptic encephalopathy and developmental delay, and the symptoms typically start between three and twelve months of age (D'alonzo et al., 2018). The patients with *CYFIP2* variants showed signs of microcephaly and began experiencing seizures around three to six months of age (Nakashima et al., 2018). Therefore, it is conceivable that CYFIP2 may have some roles in embryonic brain development.

To examine the function of CYFIP2, we characterized expression patterns of endogenous *Cyfip2* mRNAs and proteins in embryonic mice. We then compared the survival rate, body and brain size, and cortical cytoarchitecture of *Cyfip2*^−/−^ embryonic mice with those of wild-type (WT) or *Cyfip2*^+/−^ littermates. We also performed transcriptomic analyses in the cortex of *Cyfip2*^+/−^ and *Cyfip2*^−/−^ embryonic mice. The results suggest that CYFIP2 is critical for embryonic body growth and for early postnatal survival, but loss of its expression does not cause severe defects either in embryonic brain size and cortical cytoarchitecture. However, at the molecular level, expression of extracellular matrix (ECM)-related genes is altered in the cortex of *Cyfip2*^−/−^ embryonic mice.

## Materials and Methods

More information about materials and methods is provided in Supplementary Materials.

### Mice

The *Cyfip2*-mutant mice used in this study have been described previously (Han et al., 2015). The WT and *Cyfip2* mice were bred and maintained on a C57BL/6J background, and all mice used in experiments were obtained by heterozygous mating (*Cyfip2*^+/−^ X *Cyfip2*^+/−^) according to the Korea University College of Medicine Research Requirements. All procedures were approved by the Committees on Animal Research at Korea University College of Medicine (KOREA-2016-0066). The mice were fed *ad libitum* and housed under a 12 h light-dark cycle.

## Results and Discussion

**Figure 1:**
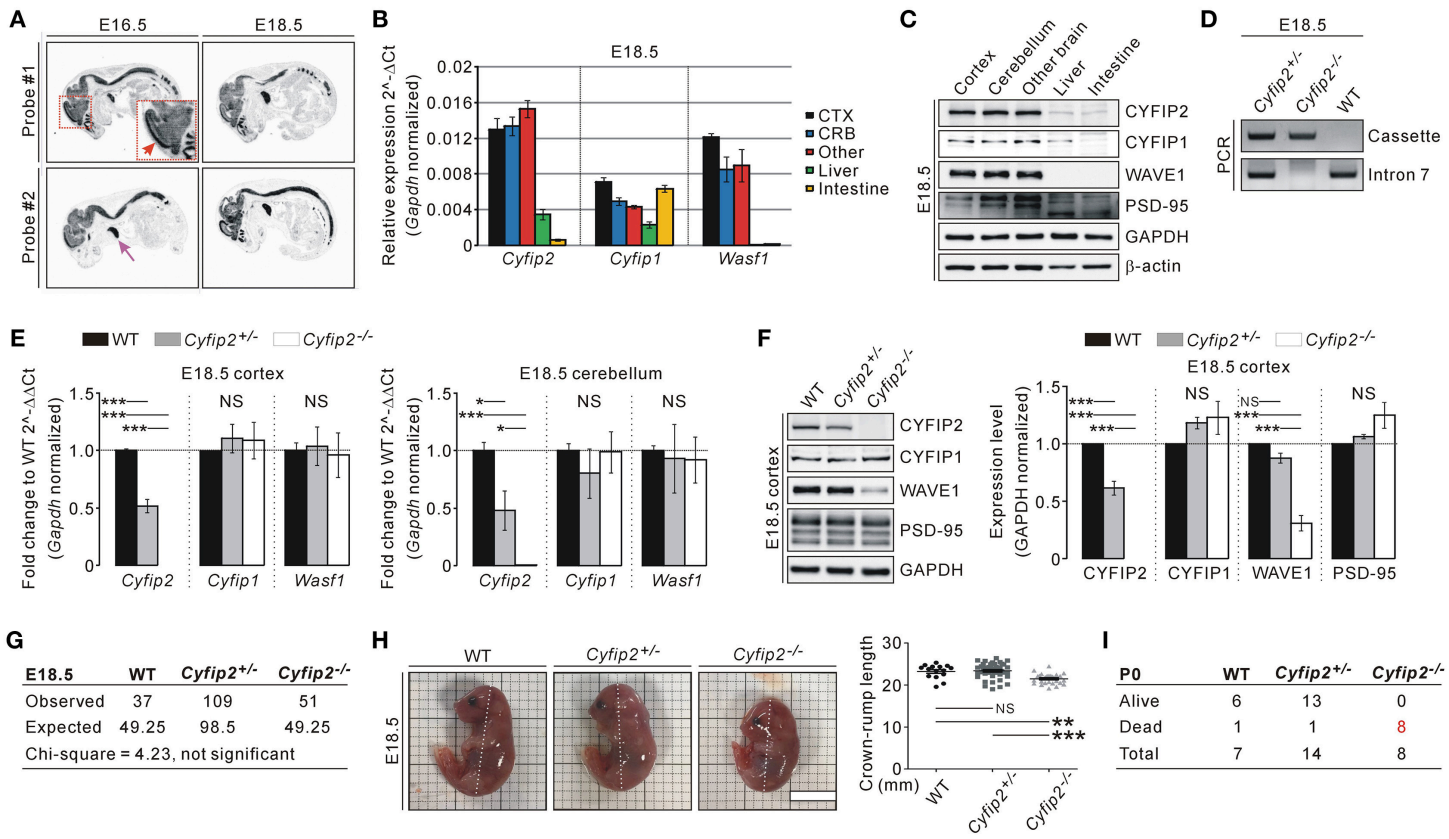
Smaller body size and early postnatal lethality of *Cyfip2*^−/−^ embryonic mice. **(A)** *in situ* hybridization analysis of *Cyfip2* in E16.5 and E18.5 WT mice. The red and purple arrows indicate the cortical region and thymus, respectively. **(B)** qRT-PCR analysis of *Cyfip1, Cyfip2*, and *Wasf1* mRNAs in tissue samples from E18.5 WT mice. CTX, cortex; CRB, cerebellum; Other, other brain regions. **(C)** Western blot analysis of CYFIP1, CYFIP2, and WAVE1 proteins in tissue samples from E18.5 WT mice. **(D)** Genotyping PCR results of E18.5 WT, *Cyfip2*^+/−^, and *Cyfip2*^−/−^ mice. **(E)** qRT-PCR analysis of *Cyfip1, Cyfip2*, and *Wasf1* mRNAs in the cortex (left panel) and cerebellum (right panel) of E18.5 WT, *Cyfip2*^+/−^, and *Cyfip2*^−/−^ mice (*n* = 4 mice per genotype). NS, not significant. **(F)** Western blot analysis of CYFIP1, CYFIP2, and WAVE1 proteins in the cortex of E18.5 WT, *Cyfip2*^+/−^, and *Cyfip2*^−/−^ mice (*n* = 4). A synaptic protein PSD-95 was blotted as a control. **(G)** The numbers of observed WT, *Cyfip2*^+/−^, and *Cyfip2*^−/−^ mice at E18.5. They are not statistically different from the numbers expected from Mendelian ratios, based on the Chi-square test. **(H)** Representative images and quantifications show smaller body size (crown-rump length, dotted line) of E18.5 *Cyfip2*^−/−^ mice compared with WT and *Cyfip2*^+/−^ littermates (*n* = 15, 44, 26 for WT, *Cyfip2*^+/−^, and *Cyfip2*^−/−^ mice, respectively). Scale bar, 1 cm. **(I)** The numbers of observed alive or dead WT, *Cyfip2*^+/−^, and *Cyfip2*^−/−^ mice at P0.

To understand the expression pattern of endogenous *Cyfip2* in embryonic mice, we performed *in situ* hybridization analysis with two independent probes against *Cyfip2* transcripts. In E16.5 and E18.5 WT mice, *Cyfip2* transcripts were detected throughout the central nervous system, and relatively strong signals were observed in the cortex (Figure 1A). Intriguingly, strong *Cyfip2* expression was also detected in the thymus but not in other organs. Consistent with the *in situ* hybridization analysis, qRT-PCR analysis showed that *Cyfip2* and *Wasf1* (encoding WAVE1) transcripts were more abundant in the brain compared with the liver and intestine of E18.5 mice (Figure 1B). Meanwhile, *Cyfip1* transcripts in the intestine were as abundant as those in the brain. At the protein level, CYFIP1, CYFIP2, and WAVE1 were expressed in the E18.5 brain at levels higher than those in the liver and intestine (Figure 1C). We observed similar results from tissue samples of postnatal day 0 (P0) mice (Figure S1).

By using two primer sets (one for the targeting cassette of *Cyfip2*-mutant mice, and the other for intron 7 of *Cyfip2*), we could identify WT, *Cyfip2*^+/−^, and *Cyfip2*^−/−^ mice at E18.5 (Figure 1D). We could confirm that *Cyfip2* mRNAs and proteins in the cortex and cerebellum were reduced by approximately 50% in *Cyfip2*^+/−^ mice compared with WT mice, and not detected in *Cyfip2*^−/−^ mice at E18.5 (Figures 1E,F). There was no change in *Cyfip1* and *Wasf1* mRNA levels in either *Cyfip2*^+/−^ or *Cyfip2*^−/−^ embryonic mice compared with WT littermates (Figure 1E). However, at the protein level, WAVE1, but not CYFIP1, was reduced in *Cyfip2*^−/−^ cortex at E18.5 (Figure 1F), which is consistent with previous reports showing decreased WAVE1 protein stability without CYFIP (Zhao et al., 2013; Han et al., 2015).

Next, we counted the numbers of WT, *Cyfip2*^+/−^, and *Cyfip2*^−/−^ mice at E18.5 to understand whether *Cyfip2*^−/−^ mice show prenatal lethality. However, from the total number of 197 mice counted, the numbers of WT, *Cyfip2*^+/−^, and *Cyfip2*^−/−^ embryos were not statistically different from the numbers expected from Mendelian inheritance ratios (Figure 1G). Nevertheless, when we measured crown-rump length of the embryos, we could find that *Cyfip2*^−/−^ mice were significantly smaller than WT and *Cyfip2*^+/−^ littermates at E18.5 (92.4% of WT, and 91.8% of *Cyfip2*^+/−^) (Figure 1H). In contrast, body weights were comparable among WT, *Cyfip2*^+/−^, and *Cyfip2*^−/−^ mice at E18.5 (Figure S2), suggesting that smaller size of *Cyfip2*^−/−^ mice was likely due to their defects in body curvature. We also counted the numbers of WT, *Cyfip2*^+/−^, and *Cyfip2*^−/−^ mice at P0. During the counting, we often observed dead pups in the cages. Indeed, after genotyping PCR, we found that all identified *Cyfip2*^−/−^ mice were dead at P0 (Figure 1I).

**Figure 2:**
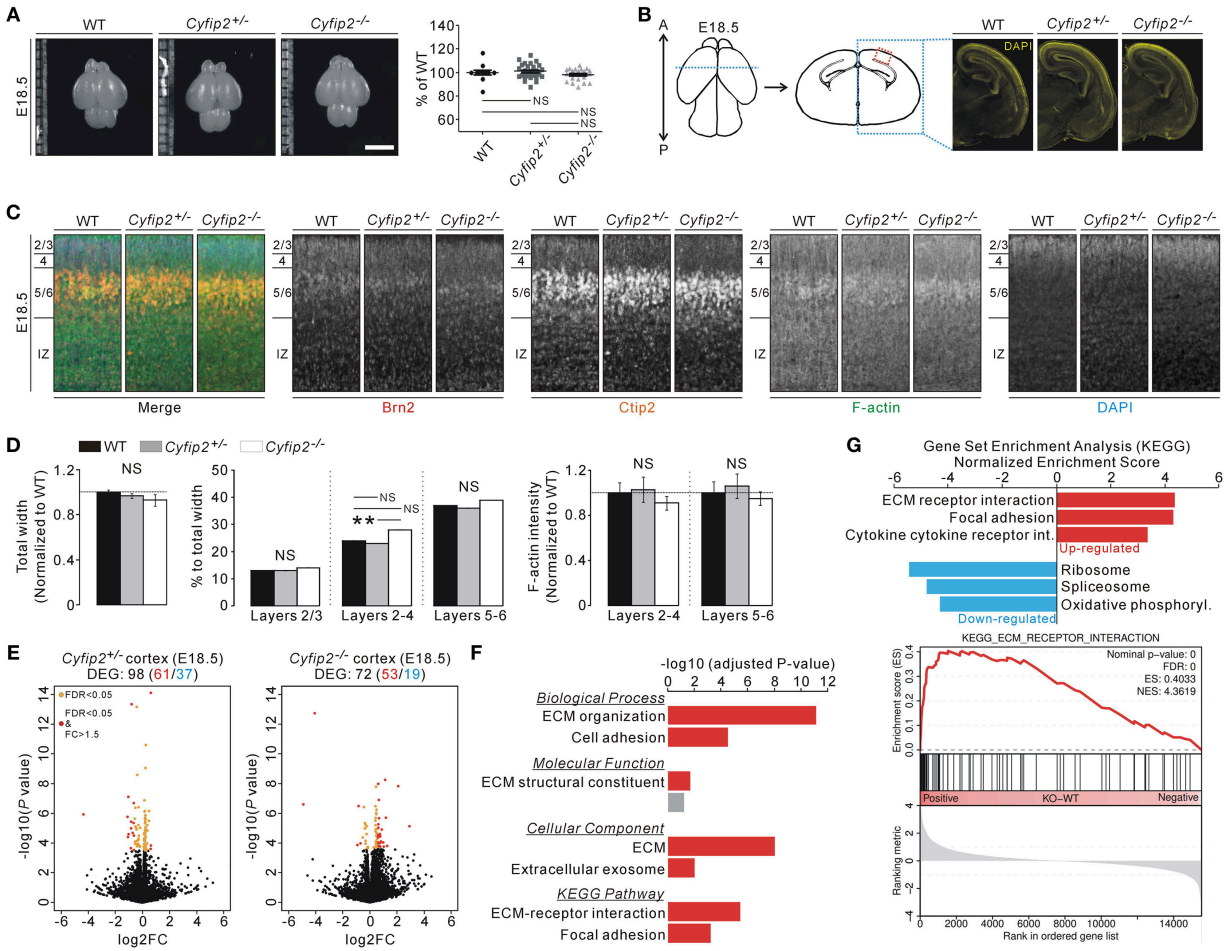
Normal brain morphology, but cortical ECM-related gene expression changes of *Cyfip2*^−/−^ embryonic mice. (**A)** Representative images and quantifications show normal brain size of E18.5 *Cyfip2*^−/−^ mice compared with WT and *Cyfip2*^+/−^ littermates (*n* = 12, 31, 20 for WT, *Cyfip2*^+/−^, and *Cyfip2*^−/−^ mice, respectively). NS, not significant. Scale bar, 3 mm. **(B)** Schematic diagrams show the regions of interests (ROIs) for immunohistochemistry analysis (left panel). Representative images of DAPI staining show that overall brain morphologies are comparable among E18.5 WT, *Cyfip2*^+/−^, and *Cyfip2*^−/−^ mice (right panel). A, anterior; P, posterior. **(C)** Representative images of Brn2, Ctip2, F-actin, and DAPI staining in E18.5 WT, *Cyfip2*^+/−^, and *Cyfip2*^−/−^ cortex (the ROI is depicted in **(B)**, red box). IZ, intermediate zone. **(D)** Quantifications of the cortical cytoarchitecture in E18.5 WT, *Cyfip2*^+/−^, and *Cyfip2*^−/−^ mice (*n* = 12 sections from 6 WT, 14 sections from 7 *Cyfip2*^+/−^, and 11 sections from 6 *Cyfip2*^−/−^ mice). **(E)** Volcano plots for the cortical RNA-seq analyses of *Cyfip2*^+/−^ (left panel) and *Cyfip2*^−/−^ (right panel) mice, respectively. DEGs, defined by FDR < 0.05, are shown as orange (FC = < 1.5) and red (FC > 1.5) circles. FC, fold change. **(F)** GO and KEGG pathway analyses of the DEGs of the *Cyfip2*^−/−^ embryonic cortex. **(G)** The bar graph shows normalized enrichment scores of GSEA on the KEGG gene sets for the cortical RNA-seq analysis of *Cyfip2*^−/−^ embryonic mice. The enrichment plot of cortical RNA-seq analysis of *Cyfip2*^−/−^ embryonic mice on the “ECM receptor interaction” gene set (lower panel).

The smaller body size and early postnatal lethality of *Cyfip2*^−/−^ embryos prompted us to investigate brain abnormalities of the mice. However, brain size, as measured by width and length of the cortical region, was comparable among WT, *Cyfip2*^+/−^, and *Cyfip2*^−/−^ mice at E18.5 (Figure 2A). Moreover, DAPI staining of the brain sections showed generally normal morphology of *Cyfip2*^−/−^ brains at E18.5 (Figure 2B). To further analyze the details of the cortical cytoarchitecture of the embryos, we performed fluorescent immunohistochemistry on the cortical area using antibodies against Brain-2 (Brn2, marker for layers 2/3, and 5) and COUP-TF-interacting protein 2 (Ctip2, marker for layer 5/6) (Figure 2C). We found that total width of the cortex (from layer 2/3 to intermediate zone), and relative width of each layer to total width (percentage of total width) were similar among WT, *Cyfip2*^+/−^, and *Cyfip2*^−/−^ mice at E18.5 (Figure 2D). We further confirmed these results with additional antibodies against Cut like homeobox 1 (Cux1, marker for layer 2/3) and T-box brain protein 1 (Tbr1, marker for layer 6) (Figure S3). However, the relative width of layers 2-4 to the total width was significantly larger in *Cyfip2*^−/−^ embryos than in the *Cyfip2*^+/−^ littermates. We also examined F-actin levels in the cortex, but they were not different among WT, *Cyfip2*^+/−^, and *Cyfip2*^−/−^ embryos. Furthermore, the total neurite lengths of WT and *Cyfip2*^−/−^ cultured cortical neurons (at days *in vitro* 4) were comparable, but those between *Cyfip2*^+/−^ and *Cyfip2*^−/−^ neurons were slightly, but significantly different (Figure S4).

Next, we performed transcriptomic analyses (RNA-sequencing [RNA-seq]) of cortical tissue from E18.5 WT, *Cyfip2*^+/−^, and *Cyfip2*^−/−^ mice, to identify any molecular changes (Table S1). After applying adjusted *P* values to the analyses, we identified 98 and 72 differentially expressed genes (DEGs) in the *Cyfip2*^+/−^ and *Cyfip2*^−/−^ cortex, respectively, compared with the WT cortex (Figure 2E and Tables S2, S3). Gene ontology (GO) and Kyoto Encyclopedia of Genes and Genomes (KEGG) pathway analyses of the 98 DEGs in the *Cyfip2*^+/−^ cortex showed no significant term in any category, possibly because of heterogeneity of the DEGs. However, same analyses on the 72 DEGs in the *Cyfip2*^−/−^ cortex revealed extracellular matrix (ECM)-related terms to be significant (Figure 2F and Table S4). Furthermore, Gene Set Enrichment Analysis (GSEA), which is used to identify molecular signatures based on the broader expression changes in the transcriptome, also suggested “ECM receptor interaction” as a significantly enriched term especially from the up-regulated genes in the *Cyfip2*^−/−^ cortex (Figure 2G and Table S5).

Taken together, our results suggest that CYFIP2 is critical for embryonic body growth and for early postnatal survival. CYFIP2 is not essential for overall embryonic brain development, in terms of gross morphology, as assessed by brain size and cortical cytoarchitecture of *Cyfip2*^−/−^ mice. However, at the molecular level, ECM-related genes are significantly altered in the cortex of *Cyfip2*^−/−^ embryonic mice. ECM affects many aspects of brain development, ranging from neuronal migration to synapse formation (Barros et al., 2011). Therefore, further detailed analyses about the cortical ECM in *Cyfip2*^−/−^ mice may potentially provide better insight toward understanding the functions of CYFIP2 in embryonic mice. However, our results may not be directly implicated in the pathophysiology that underlies early-onset epileptic encephalopathy and developmental delay associated with West syndrome, because the *CYFIP2* variants found in patients have gain-of-function effects on the WRC and do not affect CYFIP2 stability (Nakashima et al., 2018).

## Author Contributions

YZ, YL, YK, BL, JK, CJ, SK and KH designed and performed the experiments. HKa, HKi, and KH analyzed and interpreted the data. HKa and KH wrote the paper. All authors read and approved the manuscript.

### Conflict of Interest Statement

The authors declare that the research was conducted in the absence of any commercial or financial relationships that could be construed as a potential conflict of interest.
