# Supplementary material for: Smaller Body Size, Early Postnatal Lethality, and Cortical Extracellular Matrix-Related Gene Expression Changes of Cyfip2-Null Embryonic Mice
Source: Front Mol Neurosci. 2019 Jan 4;11:482. doi: 10.3389/fnmol.2018.00482 (PMC6338024; doi:10.3389/fnmol.2018.00482)
Supplement: Supplementary file 1 [file Data_Sheet_1.docx]

**Supplementary material**

**Smaller body size, early postnatal lethality, and cortical extracellular matrix-related gene expression changes of *Cyfip2*-null embryonic mice**

Yinhua Zhang, Hyojin Kang, Yeunkum Lee, Yoonhee Kim, Bokyoung Lee, Jin Yong Kim, Chunmei Jin, Shinhyun Kim, Hyun Kim and Kihoon Han

**Supplementary figures**

**
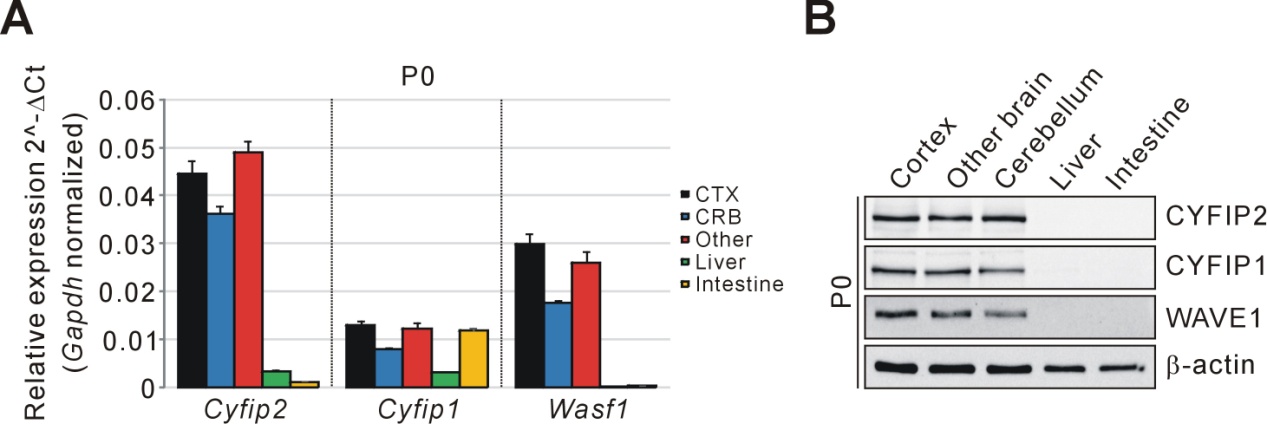
**

**Figure S1. mRNA and protein expression analyses of *Cyfip1*, *Cyfip2*, and *Wasf1* in tissue samples from P0 mice. (A)** qRT-PCR analysis of *Cyfip1*, *Cyfip2*, and *Wasf1* mRNAs in tissue samples from postnatal day 0 (P0) wild-type (WT) mice. CTX, cortex; CRB, cerebellum; Other, other brain regions. **(B)** Western blot analysis of CYFIP1, CYFIP2, and WAVE1 proteins in tissue samples from P0 WT mice.

**
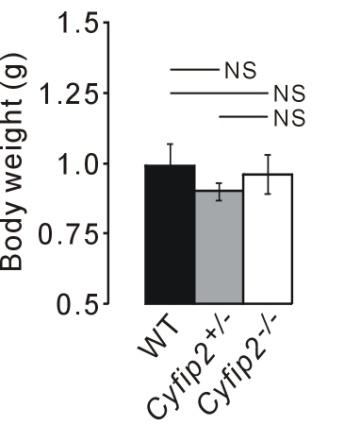
**

**Figure S2. Comparable body weights of E18.5 WT, *Cyfip2^+/-^*, and *Cyfip2^-/-^* mice.** (n = 7, 20, 7 for WT, *Cyfip2^+/-^*, and *Cyfip2^-/-^* mice, respectively; one-way ANOVA with Bonferroni's post-hoc test).

**
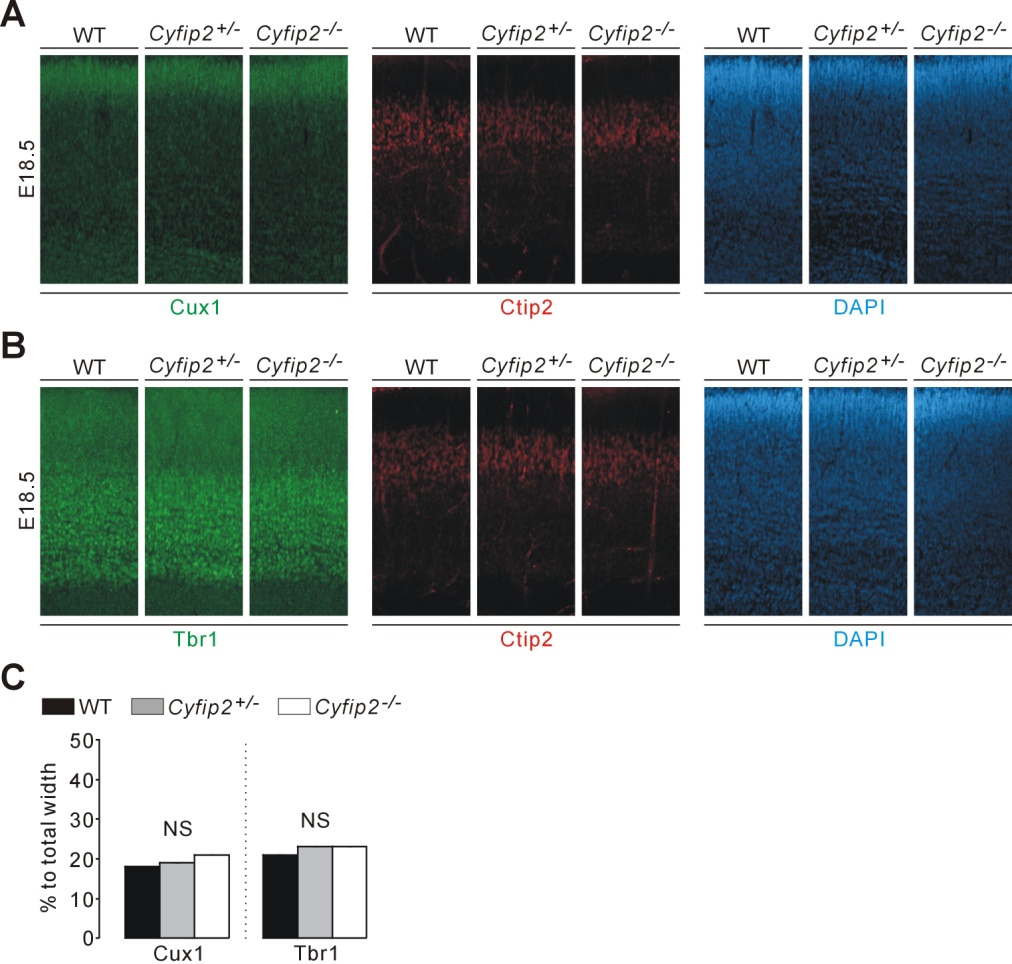
**

**Figure S3. Normal cortical cytoarchitecture of *Cyfip2^-/-^* embryonic mice. (A)** Representative images of Cux1, Ctip2, and DAPI staining in E18.5 WT, *Cyfip2^+/-^*, and *Cyfip2^-/-^* cortex (the ROI is depicted in Fig. 2B, red box). **(B)** Representative images of Tbr1, Ctip2, and DAPI staining in E18.5 WT, *Cyfip2^+/-^*, and *Cyfip2^-/-^* cortex. **(C)** Quantifications of the cortical cytoarchitecture (Cux1 for layer 2/3, and Tbr1 for layer 6) in E18.5 WT, *Cyfip2^+/-^*, and *Cyfip2^-/-^* mice (n = 12 sections from 6 WT, 10 sections from 6 *Cyfip2^+/-^*, and 7 sections from 4 *Cyfip2^-/-^* mice, respectively; one-way ANOVA with Bonferroni's post-hoc test).

**
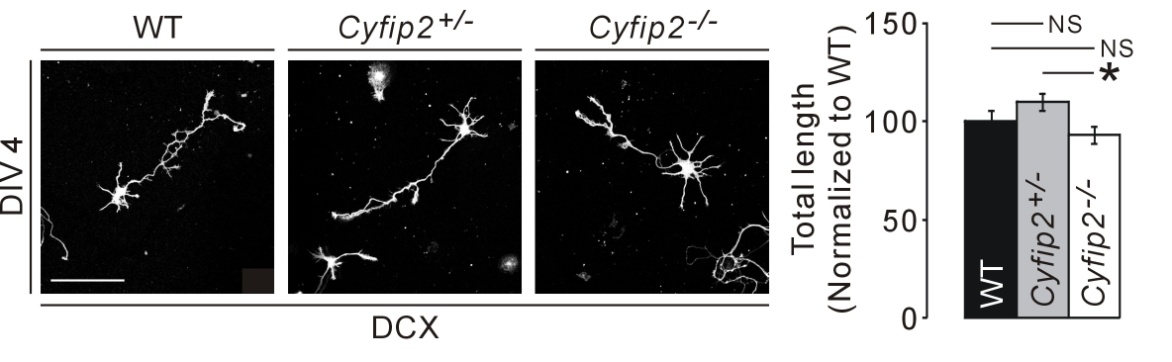
**

**Figure S4. Minimal difference in neurite outgrowth among the cultured cortical neurons of WT, *Cyfip2^+/-^*, and *Cyfip2^-/-^* mice.** Representative images and quantifications show minimal difference in the total neurite length (immunostained by Doublecortin [DCX] antibodies) among cultured cortical neurons at days in vitro (DIV) 4 of WT, *Cyfip2^+/-^*, and *Cyfip2^-/-^* mice (n = 51, 77, 47 for WT, *Cyfip2^+/-^*, and *Cyfip2^-/-^* neurons, respectively; one-way ANOVA with Bonferroni's post-hoc test).

**Supplementary materials and methods**

**In situ hybridization**

*In situ* hybridization was performed as previously described ([Kim et al., 2002](#_ENREF_5)). Hybridization probes for mouse *Cyfip2* mRNAs were prepared by cloning of the nt 3113-4063 and nt 4215-4766 of *Cyfip2* (NM_001252460.1) into pGEM-T Easy vector (Promega). Antisense probes were generated using ^35^S-UTP and the Riboprobe system (Promega).

**RNA purification and qRT-PCR**

Real-time quantitative reverse transcription PCR (qRT-PCR) was performed as described previously ([Han et al., 2013](#_ENREF_2);[Han et al., 2015](#_ENREF_1);[Kim et al., 2016](#_ENREF_6);[Lee et al., 2017a](#_ENREF_7)). Briefly, total RNA was extracted from the tissues of embryonic mice using a miRNeasy minikit (Qiagen) according to the manufacturer’s instructions. Two micrograms of total RNA was used for cDNA synthesis using the iScript™ cDNA Synthesis Kit (Bio-Rad). Target mRNAs were detected and quantified by a real-time PCR instrument (CFX96 Touch, Bio-Rad) using SYBR Green master mix (Bio-Rad). The results were analyzed using the comparative Ct method normalized against the housekeeping gene *Gapdh*. The primer sequences for real-time PCR are as follows:

Mouse *Cyfip1* forward 5’ GGTTATGGCAGGAAGTTTGC 3’,

reverse 5’ GATCGTGGCTCCCTGATTT 3’

Mouse *Cyfip2* forward 5’ AGATGTACCTGACGCCCAGT 3’,

reverse 5’ TGACATTTCCGTCCATCAGA 3’

Mouse *Wasf1* forward 5’ GAAAGTGCCAAGAGCACCTC 3’,

reverse 5’ CACGTATGTCTGTGGCCTTG 3’

Mouse *Gapdh* forward 5’ GGCATTGCTCTCAATGACAA 3’,

reverse 5’ CCCTGTTGCTGTAGCCGTAT 3’

**Biochemistry and antibodies for Western blotting**

Whole lysates of the mouse tissues were prepared as described previously ([Han et al., 2009](#_ENREF_3);[Han et al., 2015](#_ENREF_1)). Briefly, the frozen tissues of embryonic mice were homogenized in RIPA buffer (50 mM Tris-HCl pH 8.0, 150 mM NaCl, 0.1% SDS, 1% Triton X-100, 0.5% sodium deoxycholate) with freshly added protease and phosphatase inhibitors (Roche). Protein concentration was measured using the Bradford Protein Assay (Bio-Rad). Tissue lysates were heated in 1X NuPAGE LDS sample buffer (Invitrogen) containing a 1X NuPAGE reducing agent (Invitrogen). From each sample, 10~20 μg of proteins were loaded for Western blotting. The antibodies used for Western blot analysis were β-actin (Santa Cruz Biotechnology, sc-47778), CYFIP1 (Millipore, AB6046), CYFIP2 (Abcam, ab95969), GAPDH (Cell Signaling, #2118), PSD-95 (Thermo Scientific, MA1-046), and WAVE1 (NeuroMab, 75-048). Western blot images were acquired by ChemiDoc Touch Imaging System (Bio-Rad) and quantified using ImageJ software.

**Immunohistochemistry and image analysis**

Pregnant female mice were deeply anesthetized with isoflurane and sacrificed. The brains of E18.5 embryos were extracted and fixed with 4% paraformaldehyde (PFA) in phosphate-buffered saline (PBS) two overnight. After fixation, brains were washed with PBS and cryoprotected with 30% sucrose in PBS for 48 h. Frozen brains in O.C.T compound (SAKURA Tissue-Tek, 4583) were sectioned (100 μm) using a cryostat microtome (Leica, CM3050S). For antibody incubation, two coronal sections from each mouse were randomly selected at a similar anterior-posterior level. The following antibodies were used: Brn2/POU3F2 (Abcam, ab94977, 1:1000), Ctip2 (Abcam, ab18465, 1:500), Cux1 (Santa Cruz, sc-13024, 1:250), Tbr1 (Abcam, ab31940, 1:250), Alexa Flour 488-conjugated Phalloidin (Invitrogen, A12379, 1 unit/200 µl), and Alexa Fluor or Cy3-conjugated secondary antibodies (Jackson Immunoresearch, 111-606-047 and 112-165-143, 1:500). DAPI (DAPI dilactate, Invitrogen, 300 nM in PBS) was used to counterstain nucleus. Confocal microscopy (Zeiss, LSM800) was used for image acquisition (10x objective and 1x digital zoom). Whole regions were obtained by tile scanning and each frame was taken by Z-stacks of 10 slices (in total 100 µm thickness). Tiled Z-project images were aligned and turned into a single flattened image using ZEN software (Zeiss). Immunopositive signals within regions of interests (ROIs) were measured using ImageJ software. All quantifications were carried out by operators blinded to the genotype.

**Neuron culture and immunocytochemistry**

Cultured cortical neurons were prepared from E18.5 mouse brains as described previously ([Lee et al., 2017b](#_ENREF_8)). Dissociated neurons on poly-D-lysine coated coverslips (Fisher Scientific) were placed in Neurobasal medium supplemented with B27 (Invitrogen), 0.5 mM L-glutamine, and penicillin/streptomycin (Thermo Scientific). At days in vitro (DIV) 4, the neurons were fixed with 4% PFA/sucrose, permeabilized with 0.2% Triton X-100, and incubated with DCX (Doublecortin, Santa Cruz, sc-8066) and dye-conjugated secondary antibodies (Jackson ImmunoResearch). Images were acquired by confocal microscopy (Zeiss, LSM800) and quantified using ImageJ software in a blinded manner.

**RNA sequencing and analysis**

Pregnant female mice were deeply anesthetized with isoflurane and sacrificed. The cortical tissue of E18.5 embryo was dissected from each brain, immediately placed in RNAlater solution (Ambion), and stored at 4 °C overnight. A total three pairs of RNA samples (three mice per WT, *Cyfip2*^+/-^, and *Cyfip2*^-/-^) were processed for RNA sequencing. RNA extraction, library preparation, cluster generation, and sequencing were performed by Macrogen Inc. (Seoul, Korea). RNA samples for sequencing were prepared using a TruSeq Stranded mRNA LT Sample Prep Kit (Illumina) according to the manufacturer’s instructions. An Illumina’s HiSeq 2000 was used for sequencing to generate 101-bp paired-end reads (**Supplementary Table S1**). Raw data were submitted to the GEO (Gene Expression Omnibus) repository under accession number GSE119570.

Transcript abundance was estimated with Salmon (v0.11.2) ([Patro et al., 2017](#_ENREF_10)) in Quasi-mapping-based mode onto the Mus musculus genome (GRCm38) with GC bias correction (--gcBias). Quantified gene-level abundance data was imported to R (v.3.4.4) with the tximport (v1.6.0) ([Soneson et al., 2015](#_ENREF_11)) package and differential gene expression analysis was carried out using R/Bioconductor DEseq2 (v1.19.11) ([Love et al., 2014](#_ENREF_9)). The *P* values were first corrected by applying an empirical estimation of the null distribution using the R fdrtool (v.1.2.15) package and then adjusted for multiple testing with the Benjamini–Hochberg correction. Genes with an adjusted *P* value of less than 0.05 were considered as differentially expressed. Volcano plots were generated using the R ggplot2 (v.3.0.0) package.

The Gene Ontology (GO) and Kyoto Encyclopedia of Genes and Genomes (KEGG) pathway analyses were performed using DAVID software (version 6.8) ([Huang da et al., 2009](#_ENREF_4)). Mouse gene names were converted to human homologs using the Mouse Genome Informatics (MGI) database (<http://www.informatics.jax.org/homology.shtml>).

Gene Set Enrichment Analysis (GSEA) (http://software.broadinstitute.org/gsea) ([Subramanian et al., 2005](#_ENREF_12)) was used to determine whether a *priori*-defined gene sets would show statistically significant differences in expression between *Cyfip2* mutant and wild type mice. Enrichment analysis was performed using GSEAPreranked (gsea-3.0.jar) module on gene set collections H (Hallmark gene sets; 50 gene sets) and CP (KEGG; 186 gene sets) downloaded from Molecular Signature Database (MSigDB) v6.1 (http://software.broadinstitute.org/gsea/msigdb). GSEAPreranked was applied using the list of all genes expressed, ranked by the fold change and multiplied by the inverse of the *P* value with recommended default settings (1,000 permutations and a classic scoring scheme). The False Discovery Rate (FDR) was estimated to control the false positive finding of a given Normalized Enrichment Score (NES) by comparing the tails of the observed and null distributions derived from 1000 gene set permutations. The gene sets with an FDR of less than 0.05 were considered as significantly enriched.

**Quantification and statistical analysis**

Values from at least three independent experiments with biological replicates were used for quantification and statistical analysis. All quantifications were carried out in a blinded manner. *P* values were calculated by one-way analysis of variance (ANOVA) with Bonferroni's post-hoc test using GraphPad Prism 6 software. All data are present as mean ± SEM. **P*<0.05; ***P*<0.01; ****P*<0.001.

**References**

Han, K., Chen, H., Gennarino, V.A., Richman, R., Lu, H.C., and Zoghbi, H.Y. (2015). Fragile X-like behaviors and abnormal cortical dendritic spines in Cytoplasmic FMR1-interacting protein 2-mutant mice. *Human molecular genetics* 24**,** 1813-1823.

Han, K., Gennarino, V.A., Lee, Y., Pang, K., Hashimoto-Torii, K., Choufani, S., Raju, C.S., Oldham, M.C., Weksberg, R., Rakic, P., Liu, Z., and Zoghbi, H.Y. (2013). Human-specific regulation of MeCP2 levels in fetal brains by microRNA miR-483-5p. *Genes & development* 27**,** 485-490.

Han, K., Kim, M.H., Seeburg, D., Seo, J., Verpelli, C., Han, S., Chung, H.S., Ko, J., Lee, H.W., Kim, K., Heo, W.D., Meyer, T., Kim, H., Sala, C., Choi, S.Y., Sheng, M., and Kim, E. (2009). Regulated RalBP1 binding to RalA and PSD-95 controls AMPA receptor endocytosis and LTD. *PLoS biology* 7**,** e1000187.

Huang Da, W., Sherman, B.T., and Lempicki, R.A. (2009). Systematic and integrative analysis of large gene lists using DAVID bioinformatics resources. *Nature protocols* 4**,** 44-57.

Kim, H., Ha, C.M., Choi, J., Choi, E.J., Jeon, J., Kim, C., Park, S.K., Kang, S.S., Kim, K., and Lee, B.J. (2002). Ontogeny and the possible function of a novel epidermal growth factor-like repeat domain-containing protein, NELL2, in the rat brain. *Journal of neurochemistry* 83**,** 1389-1400.

Kim, Y., Zhang, Y., Pang, K., Kang, H., Park, H., Lee, Y., Lee, B., Lee, H.J., Kim, W.K., Geum, D., and Han, K. (2016). Bipolar Disorder Associated microRNA, miR-1908-5p, Regulates the Expression of Genes Functioning in Neuronal Glutamatergic Synapses. *Experimental neurobiology* 25**,** 296-306.

Lee, B., Zhang, Y., Kim, Y., Kim, S., Lee, Y., and Han, K. (2017a). Age-dependent decrease of GAD65/67 mRNAs but normal densities of GABAergic interneurons in the brain regions of Shank3-overexpressing manic mouse model. *Neuroscience letters*.

Lee, Y., Kim, D., Ryu, J.R., Zhang, Y., Kim, S., Kim, Y., Lee, B., Sun, W., and Han, K. (2017b). Phosphorylation of CYFIP2, a component of the WAVE-regulatory complex, regulates dendritic spine density and neurite outgrowth in cultured hippocampal neurons potentially by affecting the complex assembly. *Neuroreport* 28**,** 749-754.

Love, M.I., Huber, W., and Anders, S. (2014). Moderated estimation of fold change and dispersion for RNA-seq data with DESeq2. *Genome biology* 15**,** 550.

Patro, R., Duggal, G., Love, M.I., Irizarry, R.A., and Kingsford, C. (2017). Salmon provides fast and bias-aware quantification of transcript expression. *Nature methods* 14**,** 417-419.

Soneson, C., Love, M.I., and Robinson, M.D. (2015). Differential analyses for RNA-seq: transcript-level estimates improve gene-level inferences. *F1000Research* 4**,** 1521.

Subramanian, A., Tamayo, P., Mootha, V.K., Mukherjee, S., Ebert, B.L., Gillette, M.A., Paulovich, A., Pomeroy, S.L., Golub, T.R., Lander, E.S., and Mesirov, J.P. (2005). Gene set enrichment analysis: a knowledge-based approach for interpreting genome-wide expression profiles. *Proceedings of the National Academy of Sciences of the United States of America* 102**,** 15545-15550.
